# Intensity-Modulated Photoluminescence Spectroscopy for Revealing Ionic Processes in Halide Perovskites

**DOI:** 10.1021/acsenergylett.5c01102

**Published:** 2025-06-10

**Authors:** Sarah C. Gillespie, Agustin O. Alvarez, Jarla Thiesbrummel, Veronique S. Gevaerts, L.J. Geerligs, Bruno Ehrler, Gianluca Coletti, Erik C. Garnett

**Affiliations:** † LMPV-Sustainable Energy Materials Department, 55952 AMOLF Institute, Science Park 104 Amsterdam, 1098XG, The Netherlands; ‡ 2859 TNO Department Solar Energy, Westerduinweg 3, Petten 1755LE, The Netherlands; § School of Photovoltaic and Renewable Energy Engineering, 7800 University of New South Wales, Sydney, New South Wales 2052, Australia; ∥ University of Amsterdam, Science Park 904, Amsterdam, 1098XH, The Netherlands

## Abstract

Mobile ions limit halide perovskite device performance, yet quantifying ionic properties remains challenging. Frequency-domain electrical techniques are restricted to operational devices, and the resulting signals are often dominated by interfacial recombination which obscures ionic contributions. Here, we introduce intensity-modulated photoluminescence spectroscopy (IMPLS) as a fully optical alternative, where the amplitude and phase of the photoluminescence intensity is measured as a function of excitation modulation frequency. IMPLS is demonstrated on a Cs_0.07_(FA_0.83_MA_0.17_)_0.93_Pb­(I_0.83_Br_0.17_)_3_ film. Fitting the data with an optical equivalent circuit model reveals two characteristic lifetimes: τ_char_ = 2.1 ms and 77 s, likely corresponding to defect formation and ionic diffusion, respectively. The diffusion feature is consistent with intensity-modulated photocurrent/photovoltage spectroscopy (IMPS/IMVS) measurements on corresponding full devices. Importantly, IMPLS enables contact-free characterization of slow processes for all perovskite sample types, including films and devices, significantly expanding the techniques available for understanding mobile ions in these materials.

## Graphical abstract

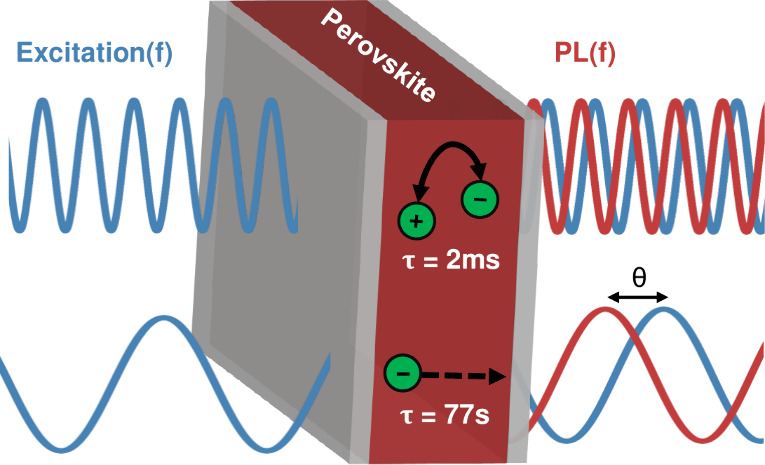

Perovskite solar cells have shown rapid improvement in power conversion efficiency, reaching values comparable to those of silicon solar cells.^1,2^ However, their widespread commercialization is still hindered, predominantly due to the instability of the perovskite material itself.^3,4^ While many of the external instability issues, such as heat- and moisture-induced degradation, can largely be prevented through effective device engineering and encapsulation strategies,^5–8^ the intrinsic perovskite instability, arising from ion migration within the perovskite film and ionic reactions at the interfaces, cannot be so easily mitigated.^9–12^ At the same time, this ionic reactivity is often reversible and not always detrimental to the overall device performance. Ionic reversibility can often lead to “perovskite healing” and even performance enhancements in some cases.^13–15^ Moreover, the influence of moving ions is pivotal in the wider application of perovskite materials in other technologies, such as in transistors, artificial synapses and self-tracking solar concentrators.^16–19^ Ionic effects can be observed over a large range of time scales, from milliseconds to hours or even years; understanding the influence of ions is therefore crucial for optimizing stability while also exploiting the self-healing effects in perovskite solar cells, LEDs and all other perovskite applications.^20–22^

There are various electrical techniques, both in the time and frequency domains, to characterize ions in metal halide perovskites. Probably the most well-known technique in the frequency domain is impedance spectroscopy (IS), with additional methods including intensity-modulated photovoltage spectroscopy (IMVS), intensity-modulated photocurrent spectroscopy (IMPS) and capacitance voltage (CV) measurements.^22–25^ Corresponding techniques in the time domain include transient (photo)­current, transient (photo)­voltage and transient capacitance techniques.^26–28^ However, each of these methods, indeed, all electrical characterization methods, are inherently limited by their requirement for electrical contacts, restricting their applicability to operational devices. The electrical response can be strongly influenced by the contacts and interfaces, making it challenging to disentangle the intrinsic properties of the perovskite film from the overall device measurements.^20,28,29^ Recently, it has been shown that combining these techniques on transport-layer free devices may elucidate properties such as mobile ion densities and activation energies, but chemical reactions and electrical effects at the contacts still remain.^30^ Furthermore, these methods are unsuitable for nonelectrical perovskite technologies, such as phosphors for next-generation LEDs and down-conversion systems.^31,32^ These techniques similarly cannot be applied as in-line characterization tools for each step of device fabrication processes in pilot lines and factories.^33^

Alternatively, there are various purely optical characterization techniques which can be applied to study perovskite thin films, half-stacks of devices, nanocrystals and other samples. One of the most popular among these techniques is time-resolved photoluminescence spectroscopy (TRPL), where the PL decay is tracked immediately after an optical excitation pulse. However, due to constraining factors in typical TRPL systems, including that the repetition rates are typically limited to high frequencies in single photon counting set-ups, this method is only useful for measuring relatively fast processes, such as electronic carrier recombination and diffusion, rather than capturing slower ionic processes or chemical reactions.^34–36^ Even using TRPL systems with a high dynamic range, such as gated charge-coupled devices, the captured carrier lifetimes have thus far only been on the order of hundreds of microseconds, much shorter than typical characteristic lifetimes of ionic processes.^21,22,35^ PL changes can also be tracked in time under continuous illumination which can provide qualitative insight into, for example, iodide interstitial migration rates and halide phase segregation rates in mixed halide systems. However, over the PL time-series, it is difficult to isolate and quantify any single process; understanding the various entangled processes which overlap in time typically requires several supplemental techniques.^37,38^

In this work, we introduce and validate a purely optical measurement technique in the frequency domain that enables the characterization of slow processes particularly in metal halide perovskites. We call this method intensity-modulated photoluminescence spectroscopy (IMPLS). Just as impedance spectroscopy is considered the frequency-domain counterpart to transient current or transient voltage measurements, or as IMPS is to transient photocurrent, IMPLS can be viewed as the frequency-domain analog of TRPL and PL time-series measurements.^25,26^

Modulated methods (IS, IMVS, IMPS and more) broadly rely on applying an input signal to the device consisting of a small sinusoidal perturbation (AC) superimposed on a steady background (DC). The amplitude of the response signal and the relative phase shift between the input and response are then measured across relevant frequencies. The main difference between each of the modulated techniques is simply which property is perturbed and which is measured.^39^ These methods are compared in Table S1 in the Supporting Information (SI). In IMPLS, the input signal is optical excitation above the sample’s bandgap. The excitation is provided by a light source with a fixed background intensity, ϕ̅_exc_, and a small modulated intensity, ϕ̃_exc_. The total illumination intensity is then:

$$\phi_{\mathrm{exc}}={\mathrm{\phi ̅}}_{\mathrm{exc}}+{\mathrm{\phi ̃}}_{\mathrm{exc}}$$ 1

The measured property in IMPLS is the photoluminescence emission from the sample, ϕ_em_, which similarly consists of both an AC and a DC component:

$$\phi_{\mathrm{em}}={\mathrm{\phi ̅}}_{\mathrm{em}}+{\mathrm{\phi ̃}}_{\mathrm{em}}$$ 2

During a measurement, both the excitation and PL signals are tracked in time for a number of cycles at a fixed frequency, *f*. From the tracked data, the DC offsets and the amplitudes of both the excitation and emission signals (|ϕ̃_exc_|, |ϕ̃_em_|), along with the relative phase shift between these signals (θ), can be determined. This measurement is then repeated over the entire relevant frequency range. The parameters are visualized in Figure 1a.

**Figure 1:**
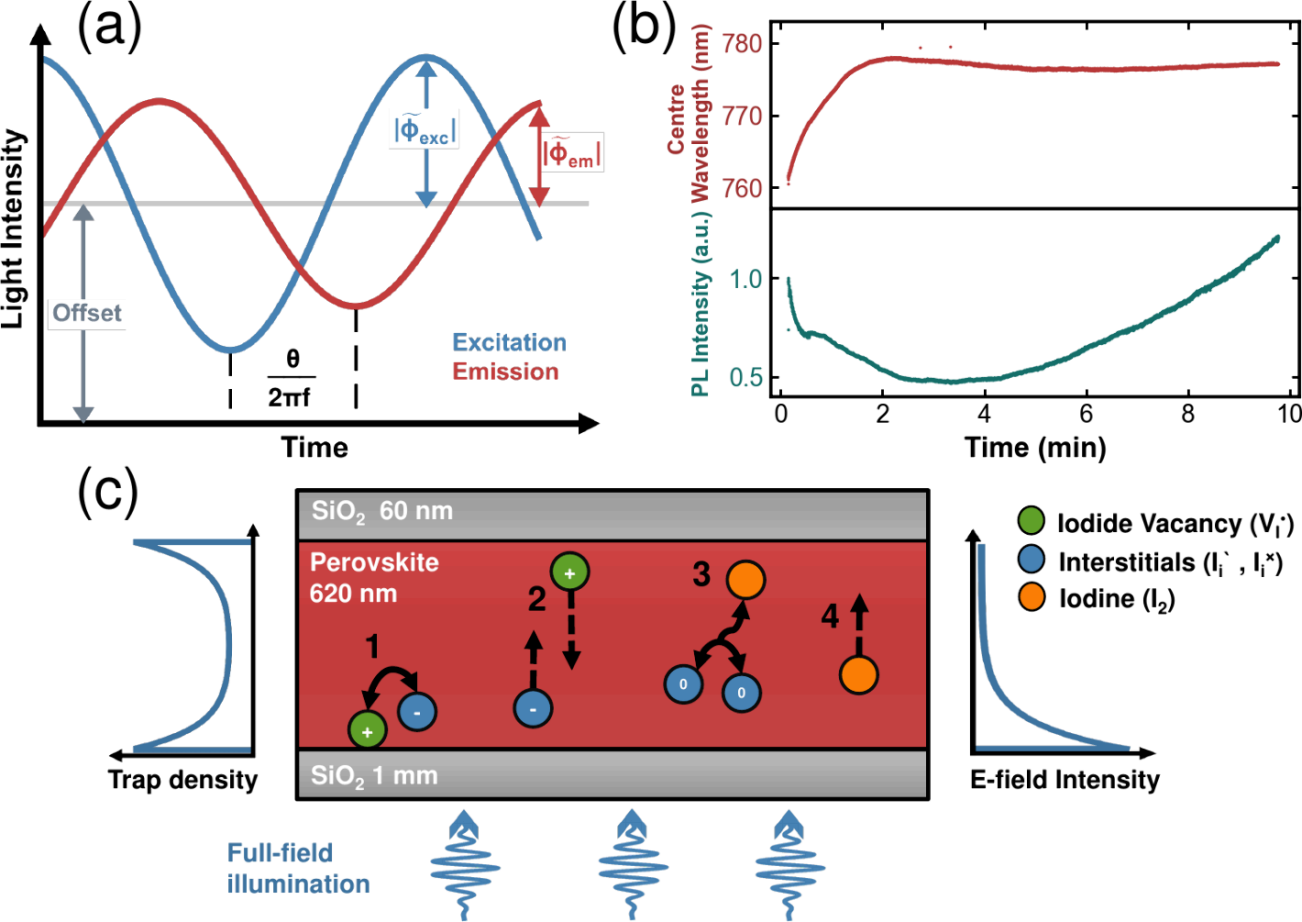
(a) Conceptual illustration of IMPLS. The schematic shows the tracking of excitation (blue) and emission (red) intensities over time at an arbitrary frequency. The key extracted parameters include the DC offset intensity for both signals, their amplitudes (|ϕ̃_exc_|, |ϕ̃_em_|), and the relative phase shift (θ) between them. For readability, the DC offsets are scaled to the same intensity value. (b) Tracking of the PL center-wavelength (top) and integrated PL intensity (bottom) of a SiO_2_-encapsulated Cs_0.07_(FA_0.83_MA_0.17_)_0.93_Pb­(I_0.83_Br_0.17_)_3_ thin film under continuous illumination for 10 min. (c) Simplified schematic of various ionic processes occurring in halide perovskite films under illumination.^38,40^ (*1*) Reversible formation and recombination of *V*_*I*_^•^/*I*_*i*_ *′* Frenkel pairs; (2) diffusion of mobile ion species, where *I*_*i*_ *′* interstitials (*V*_*I*_^•^ vacancies) are repelled from (attracted to) the surface;^38^ (3) reversible formation of iodine (I_2_) via recombination of uncharged iodine interstitials (*I*_*i*_^×^);^40^ (4) iodine diffusion to the surface. Other mechanisms not depicted include atmospheric reactions and halide segregation.^37,41^

In electrically modulated methods, the data is commonly presented using either Bode plots or Nyquist plots.^22^ In Bode plots, properties such as the amplitude, phase and capacitance are plotted against the modulating frequency. In Nyquist plots, the imaginary component of the data is plotted against the real component, for which a transfer function must be defined. Here, we define the IMPLS transfer function, *P*, to be the AC equivalent of the photoluminescence quantum yield $\overset{\sim }{\left(PLQY\right)}:$ ^42^

$$P=\overset{\sim }{PLQY}=\frac{{\mathrm{\phi ̃}}_{\mathrm{em}}}{{\mathrm{\phi ̃}}_{\mathrm{exc}}}$$ 3

Expanding the transfer function to resolve the real and imaginary components results in

$$P=P^{\prime }+iP^{\prime \prime }=\frac{\left|{\mathrm{\phi ̃}}_{\mathrm{em}}\right|}{\left|{\mathrm{\phi ̃}}_{\mathrm{exc}}\right|}\exp\left(i\theta \right)=\frac{\left|{\mathrm{\phi ̃}}_{\mathrm{em}}\right|}{\left|{\mathrm{\phi ̃}}_{\mathrm{exc}}\right|}\left(\cos\theta +i\sin\theta \right)$$ 4

It is conventional in IS, IMVS and IMPS to plot the negative of the imaginary component on the *y*-axis.^43^ To maintain consistency, we present all IMPLS Nyquist plots as −*P*″ against *P*′.

Modulated PL is not a new concept; a high-frequency variation of modulated PL has previously been applied as a means to extract the minority carrier lifetime in silicon wafers, where the relative phase shift between carrier generation and relaxation depends on the carrier lifetime.^44–47^ A related technique known as frequency resolved spectroscopy has also been applied to measure the electronic lifetimes in amorphous silicon and chalcogenide glasses.^48–51^ More recently, a theoretical model was developed that enables additional parameters, such as surface recombination velocities, to be determined from modulated PL data obtained for Cu­(In_G_a)­Se_2_ (CIGS) semiconductors.^52,53^

To date, IMPLS has not been applied to investigate ionic, chemical or any other relatively slow process in metal halide perovskites, as we propose to do so here. These slow ionic and chemical effects are observable through PL time-series measurements.^38,40,54,55^ As shown in Figure 1b, we observe complex PL dynamics on a silicon-oxide (SiO_2_)-encapsulated high-performance halide perovskite thin film with a chemical composition of Cs_0.07_(FA_0.83_MA_0.17_)_0.93_Pb­(I_0.83_Br_0.17_)_3_ (sample fabrication and characterization details are listed in Section 1 of the Supporting Information). The PL dynamics observed over a period of 10 min of continuous illumination include a red-shift in the PL center-wavelength, and competing photobrightening and photodarkening processes. Proposed mechanisms for various slow PL processes include Frenkel pair formation and annihilation, ion migration, reversible interfacial passivation due to the atmosphere, reversible iodide formation within the film, formation of superoxide species, morphological influences and many more.^37,38,40,41,56–60^ As we are measuring a fully encapsulated perovskite film in a N_2_ atmosphere in this work, we do not consider atmospheric effects to play a dominant role in the measured PL behavior. Rather, these features are more likely the result of a combination of ionic defect formation, annihilation and diffusion events (Figure 1c). Since these processes can be observed with PL in the time domain, they should also be observable in the PL frequency domain. Low-frequency IMPLS should therefore enable the extraction of key ionic properties, such as diffusion rates and accumulation times.

To validate IMPLS, we performed a proof-of-concept IMPLS measurement on the same encapsulated perovskite film as described before. Figure 2a shows a schematic of the system used to measure IMPLS, which was housed inside a nitrogen-filled glovebox. A 465 nm LED was used as the excitation source and a 650 nm long-pass filter was used to block the LED light from being detected by the photodiode. The LED DC illumination power density was set to approximately 10 mW/cm^2^ and the AC modulation amplitude was set to 10% of its DC intensity. All IMPLS measurements consisted of running 15 sinusoidal waves of light emission from the LED at a fixed frequency while simultaneously recording the LED current (*j*_LED_) and photodiode current (*j*_PL_), before moving to the next frequency point. We exemplify this procedure by plotting the LED and photodiode currents for 4 of the 15 cycles at *f* = 100 Hz in Figure 2b.

**Figure 2:**
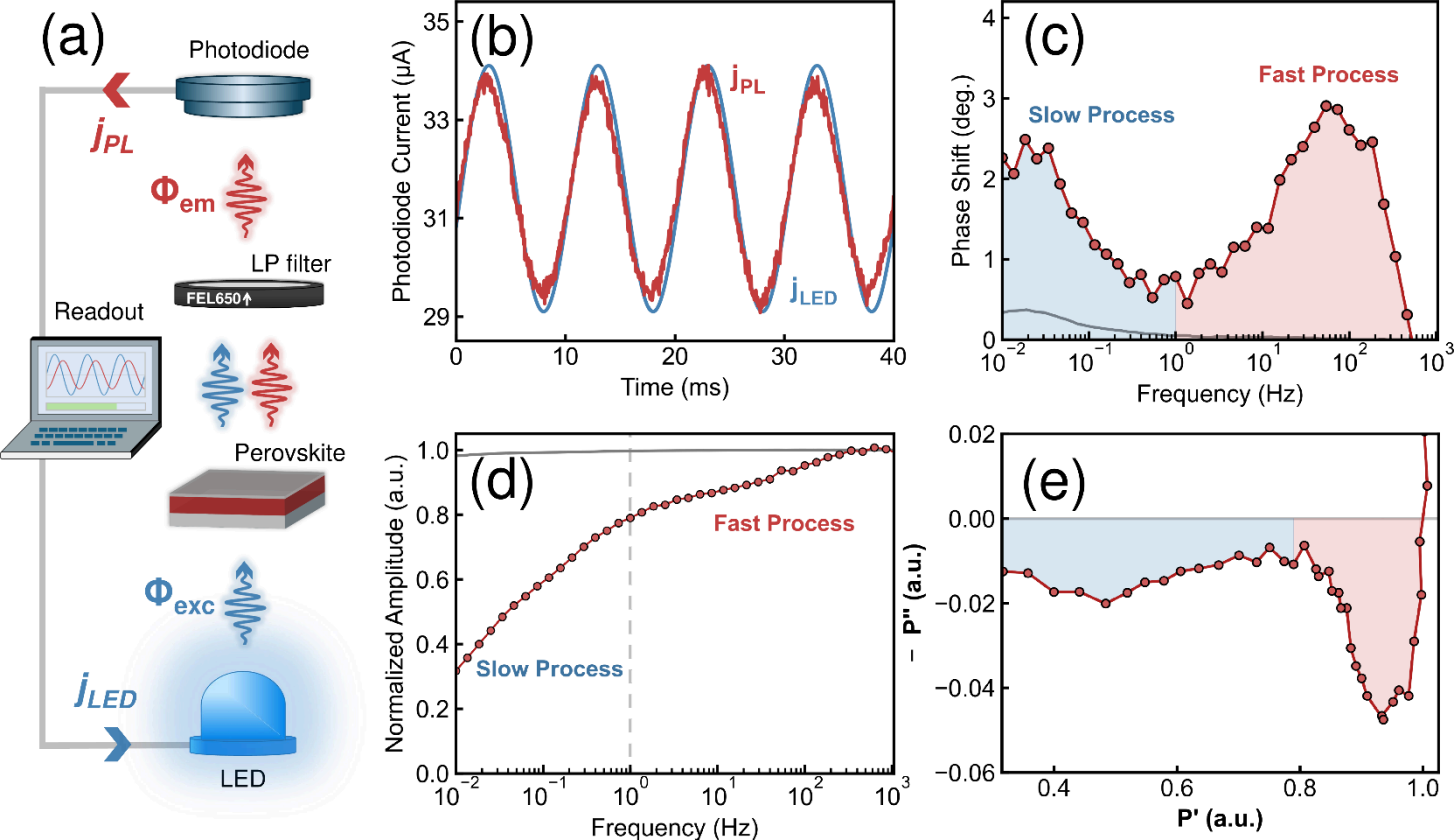
(a) The IMPLS experimental setup used in this work is housed inside a nitrogen-filled glovebox. (b) The measured photodiode current (red) resulting from the PL, plotted over the LED current (blue) for four sine wave cycles at a modulating frequency of 100 Hz. The LED current is scaled for readability. (c) The relative phase shift between the PL and the LED for frequencies ranging from 10 mHz to 1 kHz (red) and the relative phase shift of the system response (gray). (d) The corresponding amplitude of the PL intensity (red) and of the system (gray) for the same frequency range. (e) The amplitude-normalized Nyquist plot generated from the amplitude and phase data of the perovskite (note: for clarity, this exemplary Nyquist plot is not scaled 1:1; however, all subsequent Nyquist plots in this work are scaled 1:1).

Before measuring the perovskite sample, two reference measurements were performed. First, the system response was determined by measuring the photodiode current directly against the LED current (represented by the solid gray line in Figure 2c,d). Then, the extent of light leakage through the long-pass filter was determined by measuring the photodiode current against the LED current when only the long-pass filter was placed in the path between them. The results of both references for modulating frequencies up to 1 MHz are shown in SI, Figure S2. Since there is no measurable system or leakage response between 1 kHz and 1 Hz, any processes observed within this frequency range must originate from the sample. At frequencies below 1 Hz, a small phase response is measured (Figure 2c), but as the amplitude response remains unchanged in this range (Figure 2d), the dominant sample response can be distinguished from this minor system contribution.

The perovskite thin film was then measured; the results of the IMPLS measurement are shown in the phase and amplitude Bode plots in Figure 2c,d, respectively. The corresponding IMPLS Nyquist curve, calculated using eq 3, is visualized in Figure 2e. Two distinct features, shaded in red and blue in the phase plot, indicate that at least two dynamic processes are captured in this measurement. The approximate characteristic frequencies of these processes are at 100 Hz (red) and 10 mHz (blue). For now, we will refer to these processes simply as the “fast process” (100 Hz) and the “slow process” (10 mHz) in our sample. SI, Figure S3, shows that the fast process quenches under prolonged light exposure times. Therefore, to analyze the processes individually, the IMPLS measurement was separated into two parts, where the processes were independently probed and demonstrated reversibility (Figure S4). This requirement of reversibility follows from the analogy with IS, where a process must remain stable while it is probed to ensure the data can be fitted to appropriate models.^61^ More information on these measurements is available in the SI.

To quantify the observed processes, an optical equivalent circuit (OEC) model was developed and applied to separately fit the fast and slow processes. Equivalent circuit models have been used extensively in modulated electrical techniques (IS, IMPS, IMVS) to provide a deeper understanding of the underlying processes.^22,39^ Although we do not have electrical contacts, we can still use the optical emission as a proxy for the electrical circuit. This is done extensively in photovoltaics in a common measurement known as Suns-*iV*_OC_.^62–64^ In that case, the excitation intensity is a proxy for the current of the device and the PL intensity is a proxy for the voltage, since both rely on the ratio between radiative and nonradiative recombination. Here, we also equate the excitation intensity to the generated current, represented by the current generator element (blue) in Figure 3a, and use a combination of resistors and inductors across different branches to represent the various recombination processes in the perovskite.

**Figure 3:**
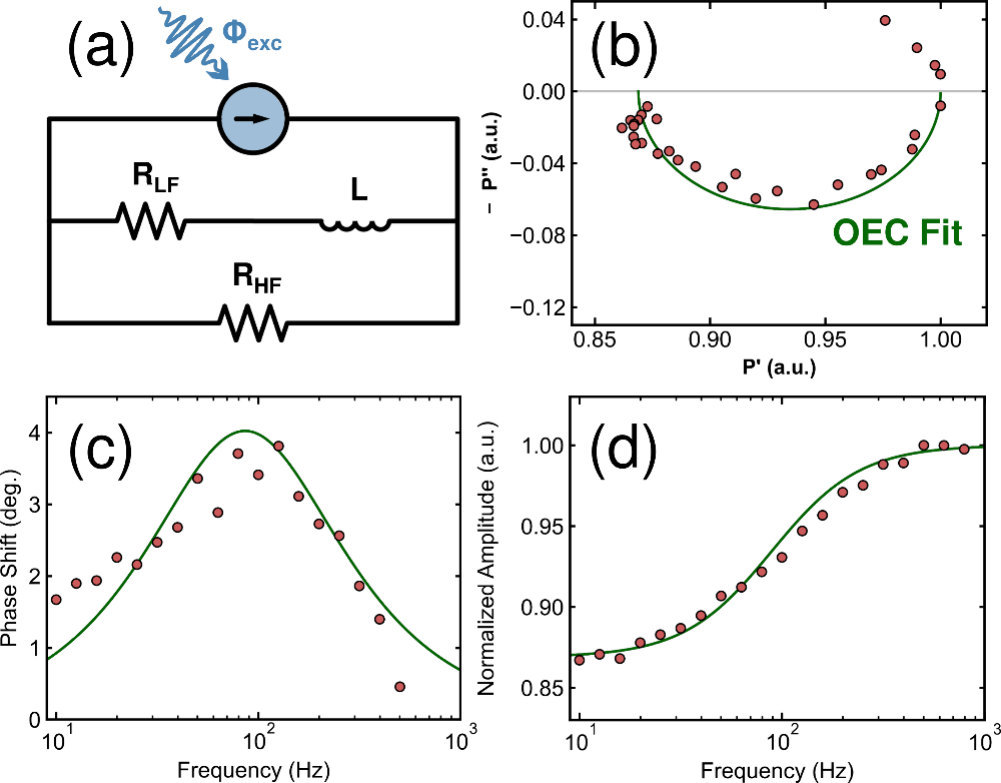
(a) The optical equivalent circuit model applied to fit the fast component of the IMPLS data. The resulting fit of the OEC model is shown in green in (b) the Nyquist plot, (c) the relative phase shift Bode plot and (d) the amplitude-normalized Bode plot.

Before applying the optical equivalent circuit model in Figure 3a to fit the IMPLS data, we will first walk through the physical interpretation of this model for different modulating frequencies. At sufficiently high frequencies, all of the “current” flows only through the high-frequency (HF) branch, which contains one resistor, *R*_HF_. This resistor represents all of the carrier recombination processes, radiative recombination, Auger recombination and trap-assisted recombination, which are frequency-independent for the frequency range of IMPLS measurements conducted in this work. In other words, the corresponding carrier lifetimes of these processes (τ_rad_, τ_Auger_, τ_trap_) are much shorter than the measurement range (denoted by the frequency range, *f*_range_) of our setup:

$$\frac{1}{2\pi f_{\mathrm{range}}}\gg \tau_{\mathrm{rad}},\tau_{\mathrm{Auger}},\tau_{\mathrm{trap}}$$ 5

This is a valid assumption as the highest frequency of interest in our measurements is *f* = 1 kHz (corresponding to a lifetime of 159 μs), and we have previously determined that the effective trap-assisted carrier lifetime of this perovskite material is approximately 3 orders of magnitude shorter, τ_trap_ ≈ 200 ns.^36^

As the modulating frequency is reduced, the “current” can also pass through the low-frequency (LF) branch. The relevant frequency at which this occurs is determined by the values of the low-frequency resistor (*R*_LF_) and of the inductor (*L*), using the relationship:

$$\tau_{\mathrm{char}}=\frac{1}{2\pi f_{\mathrm{char}}}=\frac{L}{R}$$ 6

where *f*_char_ is the characteristic frequency of the process and τ_char_ is the corresponding characteristic lifetime. Whether this frequency-dependent pathway increases or reduces the PL depends on the nature of process itself, for example, formation or annihilation of Frenkel pairs will either decrease or increase the PL, respectively.^38,56^

The OEC shown in Figure 3a was applied to fit to the IMPLS data collected for the fast process in SI, Figure S7. The amplitude was normalized so that the value of *R*_HF_ = 1, allowing for the relative losses due to the LF branch to be quantified. The fits of the Nyquist, amplitude and phase plots of the reverse scan are shown in green in Figure 3 (similar forward scan fits are shown in SI, Figure S7c,S7d). The extracted quantities of the relative resistances (in arbitrary units) and the inductance (in seconds) of this process are summarized in SI, Table S2. From these parameters, τ_char_ and *f*_char_ were determined using eq 6. Averaging the values between the forward and reverse scans, *f*_char,fast_ = 76 ± 8 Hz, corresponding to a lifetime of τ_fast_ = 2.1 ± 0.2 ms. The ratio between *R*_HF_ and *R*_LF_ provides insight into how many carriers recombine via the LF branch at sufficiently low frequencies. The ratio between these resistances is *R*_HF_/*R*_LF_ = 0.135. This indicates that 13.5% of the total carriers in the system, that would otherwise recombine radiatively at high frequencies (via *R*_HF_), recombined nonradiatively through *R*_LF_ at times longer than the characteristic lifetime of 2.1 ms.

To quantify the slow process, the OEC in Figure 4a was used to fit the blue curve shown in SI, Figure S4. Unlike the ideal inductance-like behavior of the fast process (where the Nyquist arc is a perfect semicircle), the slow process exhibited nonideal behavior. This is evident from the stretched Nyquist arc in Figure 4b.^65^ The nonideal inductor in this case is represented by *L*_α_. Otherwise, the physical interpretation of the OEC is consistent with what was previously described. By extrapolating the fit (represented by the dashed green lines in Figure 4), we extract *f*_char,slow_ = 2.1 ± 0.7 mHz, corresponding to a lifetime of τ_slow_ = 77 ± 45 s. The ratio between *R*_HF_ and *R*_LF_ for the slow process is much more substantial than the fast process, *R*_HF_/*R*_LF_ = 0.588, indicating that this is a significant loss process. The parameters obtained from the fit of the slow process are listed in SI, Table S3, and summarized in Table 1. The complete OEC diagram, which separates electronic processes from the fast and slow processes, is presented in SI, Figure S6. Further discussion of OEC modeling is provided in Section 3 of the Supporting Information.

**Figure 4:**
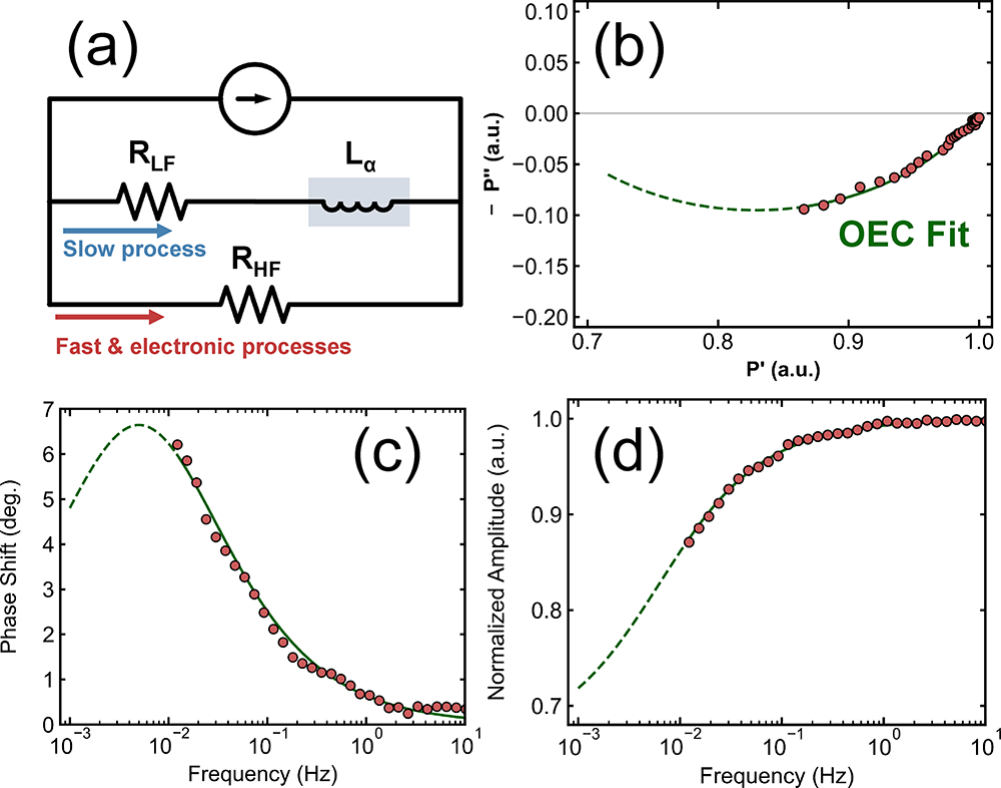
(a) The OEC applied to fit the slow component of the IMPLS data. The key difference between this OEC and the OEC applied to fit the fast process is the requirement of a nonideal inductor (highlighted in gray) to account for the stretched Nyquist arc. (b) The Nyquist plot, (c) the relative phase shift Bode plot, and (d) the amplitude-normalized Bode plot. The solid green line in these panels represents the OEC fit to the data while the dashed green line represents the extrapolation of the fit.

**Table 1:** Key Parameters Obtained from the Fits of the Relevant Optical Equivalent Circuit Models to the Fast and Slow Processes

process	*f*_char_ (Hz)	τ_char_ (s)	*R*_HF_/*R*_LF_ (%)
fast	76 ± 8	(2.1 ± 0.2) × 10^–3^	13.5
slow	(2.1 ± 0.7) × 10^–3^	77 ± 45	58.8

Considering the IMPLS-extracted characteristic frequencies, we tentatively propose that the mechanisms responsible for the fast and slow responses are likely the result of mobile ion or iodine species formation and diffusion. These mechanisms have already been proposed to describe the competing photobrightening/photodarkening PL features which have been observed in the time domain (Figure 1c).^38,40,56,58^ In previous works, it has been postulated that the underlying driving force for the diffusion of mobile ion species is a consequence of the trap-state density distribution as a function of depth in the perovskite film (Figure 1c, left).^38,56^ Under illumination, some traps – particularly near the surface – are filled by the generated electronic carriers, leading to the formation of a light-induced electric field (Figure 1c, right). We similarly assume that during an IMPLS measurement, the superimposed AC and DC illumination from the LED generates a modulating electric field. As our sample is globally illuminated by the LED, we further assume that this electric field is equally strong laterally and decays only along the transverse direction. If mobile ionic species are present in the sample (like those shown in Figure 1c), and if they diffuse through the entire film, we can calculate their diffusion coefficients using

$$D=W^{2}/\tau_{\mathrm{diff}}$$ 7

where *W* is the thickness of the film (620 nm, measured with profilometry) and τ_diff_ = τ_char_ in the case where diffusion is the rate-limiting step. The diffusion coefficient for the fast process would then be *D*_fast_ = 1.8 × 10^–6^ cm^2^/s. However, based on the literature values for even the fastest diffusive species, halide vacancies, this coefficient is still several orders of magnitude higher than expected.^20,30,66^ More likely, we anticipate that the fast process is related either to the light-induced formation of Frenkel defects, or to the formation of iodine species in the film.^38,40,56^ Both of these are loss processes and consequently result in the reduction in the PL amplitude signal. This fast process therefore corresponds to the rapid initial drop in PL seen in the time domain (Figure 1b) upon illumination.

In contrast, the calculated diffusion coefficient of the slow process is *D*_slow_ = 4.99 × 10^–11^ cm^2^/s. This value is consistent with literature values for the diffusion coefficient range for iodide vacancies in polycrystalline perovskite films, typically ranging from 10^–10^ to 10^–12^ cm^2^/s.^21,30,67^ It is therefore likely that we are observing the influence of iodide vacancies at the perovskite interface after they have diffused through the film. As the PL amplitude decreased for this process (corresponding to the slower PL reduction observed in the time domain in Figure 1b), it indicates that this interaction is a loss mechanism. This loss may result from the interaction of vacancies in the high trap-state density region at the interface, or potentially from vacancies screening other vacancies – preventing recombination with their corresponding iodide interstitials near the surface. The interpretation of the fast and slow processes as defect formation and diffusion, respectively, is also consistent with the ideal and nonideal inductive-like behaviors observed in the corresponding OEC model; the physical interpretation of the ideal and nonideal behavior is further described in the SI. However, further investigation is required to fully elucidate the exact nature of these processes.^65,68^

From the experimental validation that IMPLS can resolve slower processes in perovskite films, we now consider its potential application as a contact-free diagnostic tool to fingerprint perovskite films for latent instability. Device degradation is predominantly a consequence of mobile ions creating carrier extraction barriers at the interfaces.^9,69,70^ If IMPLS could indicate the presence of such effects prior to device fabrication, it would offer a valuable tool for optimizing both device stability and manufacturing processes. To assess this possibility, we examined whether the IMPLS responses observed above are similarly detectable in corresponding electrical measurements in perovskite solar cells. Full devices were fabricated using the same perovskite composition (Section 4 of the Supporting Information lists the device fabrication details). Figure 5a shows a representative device current density–voltage (*JV*) curve, from which a moderate device efficiency of 14.8% is calculated. The device exhibits inverted (or inductive) hysteresis which may tentatively be linked to the inductive features observed in IMPLS.^71^ Moreover, hysteresis in scan-rate-dependent *IV* measurements (SI, Figure S9) suggests that mobile ions affect the device performance.^70,72^ However, such voltage scan-rate measurements are difficult to directly link to dynamic physical processes. As a frequency-domain alternative, we replicated IMPLS-like conditions by modulating the illumination of the full device using a 450 nm LED, with modulation frequencies ranging from 10 mHz to 100 Hz. The resulting *IV* curves (Figure 5b) reveal frequency-dependent variations which are more prominent at short-circuit current, *I*_SC_, than at open-circuit voltage, *V*_OC_ (SI, Figure S9c). Like the scan-rate dependent *IV* curves, this suggests that there is an induced mobile ion screening effect which also occurs under modulated light-soaking conditions.

**Figure 5:**
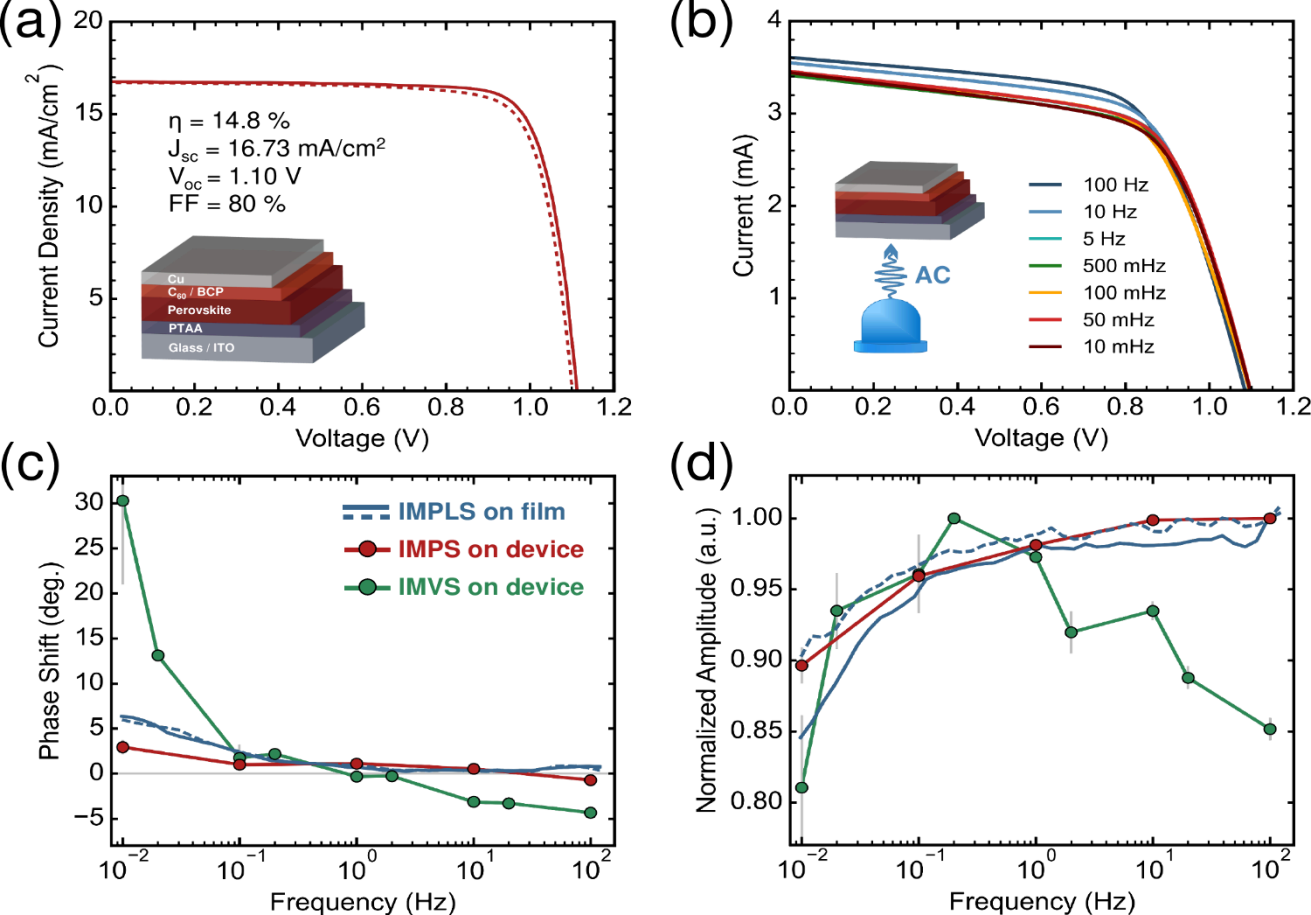
(a) *JV* curve of a representative perovskite solar cell measured under standard test conditions. The solid and dashed lines represent the forward and reverse scans, respectively. (b) Measured *IV* curves of an unmasked sample illuminated with a 450 nm LED. The *IV* curves were measured immediately after a modulated light-soaking treatment, where the LED was sinusoidally varied with modulation frequencies listed in the panel. (c) Comparison of the encapsulated perovskite film IMPLS phase shift (blue), the solar cell IMPS phase shift (red) and the solar cell IMVS phase shift (green). (d) Comparison of the normalized IMPLS amplitude (blue), IMPS amplitude (red) and IMVS amplitude (green). The light gray bars in (c) and (d) represent the associated error in the IMPS and IMVS measurements.

Given the strong current response in the device measurements, we performed IMPS at *I*_SC_, and additionally measured IMVS at *V*_OC_ (as the PL intensity is directly related to the *V*_OC_).^73^ Figure 5c,d show the phase and amplitude responses of the IMVS (green), IMPS (red), and IMPLS (blue) measurements, respectively. Notably, all three techniques reveal inductive-like behavior at frequencies below 1 Hz. The amplitude responses from IMPS and IMPLS exhibit a particularly strong correlation across the entire frequency range, with a relative amplitude difference of less than 5% between them. This suggests that the low-frequency loss process identified in IMPLS may be linked to the same mobile ion screening mechanism responsible for reduced current extraction observed in IMPS. There is similarly a correlated reduction in the IMVS amplitude at low frequencies, which is expected based on the reduction in the IMPLS amplitude. Interestingly, the IMVS amplitude trend diverges from IMPLS/IMPS above 1 Hz. This deviation is notably also present in the solar cell IMPLS amplitude response (SI, Figure S10a). The device IMPLS phase similarly follows the same trend as IMVS at relatively higher frequencies (SI, Figure S10b). These additional features signify that additional defect-related processes are occurring within the device which are absent in the symmetric SiO_2_-encapsulated film. As only the contacts are different between the film and device, the defect formation is likely related to one or both of the electrical interfaces. While further investigation is required, the observed correlations highlight the promise of IMPLS as a contact-free screening method for perovskite device stability and performance.

In conclusion, we have presented IMPLS as a fully optical technique capable of quantifying and resolving ionic and other slow processes in metal halide perovskite materials. Through experimental validation of IMPLS on a SiO_2_-encapsulated perovskite film, we demonstrated that in the 10 mHz to 1 kHz frequency range, at least two distinct loss processes occur within the material, likely corresponding to ionic species formation and mobile species diffusion to the perovskite interfaces. Additionally, we have shown that IMPLS data can be analyzed using an optical equivalent of the standard equivalent circuit model fitting procedure. The physical interpretation of this OEC model aligns with the carrier rate equation in the frequency domain, where frequency-dependent processes are represented by complex circuit components. Fitting IMPLS data to OEC models simplifies immediate data analysis, as standard EC models are already well-established in electrical analog techniques. Moreover, the interpretation of IMPLS data is simplified compared to electrical measurements as the influence of carrier extraction is eliminated, and interfacial recombination can be minimized by applying passivating contacts on either side of the film. However, IMPLS as a technique requires further benchmarking to confirm that the observed processes are indeed related to ionic species formation and diffusion, rather than other phenomena, such as slow chemical reactions at the interfaces. Temperature-dependent IMPLS measurements, combined with appropriate system modeling, could significantly enhance the interpretation and application of IMPLS in the near future.

As an outlook, IMPLS could be adapted for localized measurements using a focused LED or laser, instead of a full beam as applied here. Samples could be mapped using point-scanning or full-field imaging approaches while depth information may be resolved by varying the excitation wavelength.^34,38^ Furthermore, as the peak energy position of the PL relates to the local halide concentration in perovskites, while the width of the PL peak relates to disorder in the material, it may be even possible to obtain chemical information by measuring frequency-resolved IMPLS spectra.^37^ All of these possibilities will be explored by us in immediate follow-up research. From this foundational work, we postulate that IMPLS could eventually be applied to measure interfacial effects in halide perovskite devices by performing measurements at each step of the fabrication process, similar to sequential PLQY measurements, which are commonly used to quantify loss processes in many studies.^9,74,75^

## Supplementary Material



## Notes

Experimental methods, supporting figures, OEC analysis, OEC fit parameters. (PDF)
