# Supplementary material for: Intensity-Modulated Photoluminescence Spectroscopy for Revealing Ionic Processes in Halide Perovskites
Source: ACS Energy Lett. 2025 Jun 10;10(7):3122–31. doi: 10.1021/acsenergylett.5c01102 (PMC12261318; doi:10.1021/acsenergylett.5c01102)
Supplement: Supplementary file 1 [file nz5c01102_si_001.pdf]

Supporting Information

Intensity-Modulated Photoluminescence Spectroscopy for Revealing Ionic Processes in Halide Perovskites

Sarah C. Gillespie[1,2], Agustin O. Alvarez[1*], Jarla Thiesbrummel[1], Veronique S. Gevaerts[2], L.J. (Bart) Geerligs[2], Bruno Ehrler[1], Gianluca Coletti[3], Erik C. Garnett[1,4*]

[1] LMPV-Sustainable Energy Materials Department, AMOLF Institute, Science Park 104, Amsterdam, 1098XG, The Netherlands
[2] TNO Department Solar Energy, Westerduinweg 3, Petten, 1755LE, The Netherlands
[3] School of Photovoltaic and Renewable Energy Engineering, University of New South Wales, Sydney, New South Wales 2052, Australia
[4] University of Amsterdam, Science Park 904, Amsterdam, 1098XH, The Netherlands

* Corresponding authors. Email: a.alvarez@amolf.nl, e.garnett@amolf.nl

1. Fabrication and Characterization of Encapsulated Halide Perovskite Thin Films

The perovskite solution was prepared a nitrogen-filled glovebox by separately dissolving PbI_2 (≥99.99%, TCI) and $PbBr_2$ (≥98%, TCI) in 1.5M solutions of DMF:DMSO (4:1) and stirring at 70 °C overnight. The PbI_2 solution was added to FAI powder (≥99%, TCI) with an excess of DMF:DMSO to achieve a 1.24M solution of $FAPbI_3$ containing a 10%-molar excess of PbI_2. The $PbBr_2$ solution was added to MABr powder (≥99%, TCI) with an excess of DMF:DMSO to achieve a 1.24M solution of $MAPbBr_3$, also containing a 10%-molar excess of $PbBr_2$. The perovskite solutions, and a 1.5M solution of CsI (≥ 99.99%, Sigma-Aldrich) in DMSO, were stirred for a further 2 hours at 70 °C. The $FAPbI_3$ and $MAPbBr_3$ were then combined in an 83:17 ratio and the CsI solution was subsequently added to form the $Cs_{0.07}(FA_{0.83}MA_{0.17})_{0.93}Pb(I_{0.83}Br_{0.17})_3$ solution, and stirred again at 70 °C for a final 2 hours. Before deposition, the perovskite solution was cooled to room temperature and filtered with a 0.45 µm PTFE filter.

Glass substrates were cleaned by scrubbing with a 1% Hellmanex III solution in water, followed by three 15 minute sonication cycles: first in 70 °C water, then in acetone and finally in isopropanol. The substrates were dried under a nitrogen gas flow. Immediately before spin-coating, the clean substrates were treated with UV-Ozone for 30 minutes.
For each sample, 120 µL of the perovskite solution was deposited on the glass substrate and then spin-coated at 4000 RPM for 30 seconds, with ramp-up time of 6 seconds. 15 seconds before the end of the cycle, the sample was quenched with 170 µL of filtered Chlorobenzene (0.22 µm PTFE filter). The sample was then annealed at 100 °C on a hotplate for 30 minutes.

Finally, the perovskite samples were encapsulated in SiO_2 using a Polyteknik Flextura M508E electron-beam evaporator. 60 nm of SiO_2 was deposited under vacuum directly from a SiO_2 target at an evaporation rate of 0.06 nm/s. The surface recombination velocity between the SiO_2 and perovskite interface is 200 cm/s – substantially lower than typical interfaces applied in corresponding solar cells[1]. The SiO_2 top layer additionally acts as a barrier to prevent the evaporation of volatile perovskite components.

The perovskite film formation was confirmed using x-ray diffraction (XRD, measured with a Bruker D2 phaser with a Cu Kα tube), absorbance spectra (Perkin Elmer LAMBDA 750 UV/Vis/NIR Spectrophotometer) and photoluminescence spectra (WiTEC alpha300 SR confocal imaging microscope coupled to a Thorlabs S1FC405 405nm CW diode laser). The results of these measurements are shown in Figure S1.

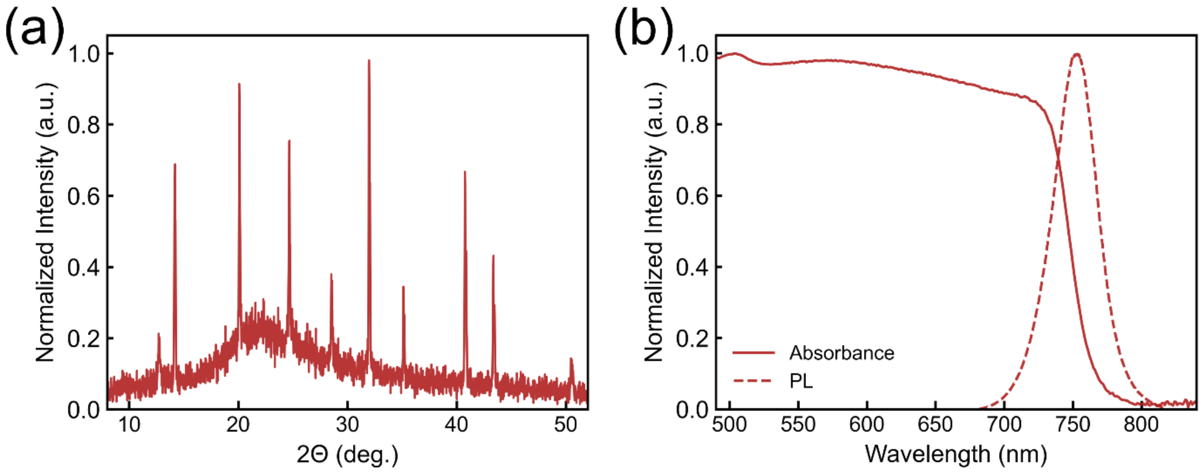

Figure S1: (a) The X-ray diffractogram of the metal halide perovskite sample following encapsulation in SiO_2 confirms the perovskite formation and correct perovskite phase. (b) The normalized absorbance curve (solid line) and PL (dashed line) of the same sample. The PL spectrum was obtained immediately after photoexcitation.

2. IMPLS Characterization

A comparison between IMPLS and the common modulated electrical and electrical-optical hybrid techniques are shown in Table S1[2,3]. Impedance spectroscopy (IS) is most commonly performed with voltage acting as the modulating parameter and current as the response, however the converse configuration (using current modulation and measuring voltage) is also possible and still regarded as IS.

Table S1: Summary of the input parameters, output parameters and the transfer functions of purely electrical, purely optical and optical-electrical modulated techniques.

Method	Abbreviation	Perturbation	Response	Transfer Function
Impedance Spectroscopy	**IS**	Voltage, $\tilde{V}$	Current, $\tilde{j}_e$	$\mathbf{Z} = \tilde{V} \;/\; \tilde{j}_e$
Intensity-Modulated Photovoltage Spectroscopy	**IMVS**	Light in, $\tilde{\phi}_{exc}$	Voltage, $\tilde{V}$	$\mathbf{W} = \tilde{V} \;/\; \tilde{\phi}_{exc}$
Intensity-Modulated Photocurrent Spectroscopy	**IMPS**	Light in, $\tilde{\phi}_{exc}$	Current, $\tilde{j}_e$	$\mathbf{Q} = \tilde{j}_e \;/\; \tilde{\phi}_{exc}$
Intensity-Modulated Photoluminescence Spectroscopy	**IMPLS**	Light in, $\tilde{\phi}_{exc}$	Light out, $\tilde{\phi}_{em}$	$\mathbf{P} = \tilde{\phi}_{em} \;/\; \tilde{\phi}_{exc}$
Voltage-Modulated Emission Spectroscopy	**VMES**	Voltage, $\tilde{V}$	Light out, $\tilde{\phi}_{em}$	$\mathbf{S} = \tilde{\phi}_{em} \;/\; \tilde{V}$
Current-Modulated Emission Spectroscopy	**CMES**	Current, $\tilde{j}_{in}$	Light out, $\tilde{\phi}_{em}$	$\mathbf{R} = \tilde{\phi}_{em} \;/\; \tilde{j}_{in}$

The IMPLS system, illustrated in Figure 2a of the main text was constructed inside a N_2-filled glovebox. The 465 nm blue LED (Cree LED) and the silicon photodiode (model BPW34, OSRAM, Infineon Technologies) were connected to a combined system-controller and system-readout unit (PAIOS from Fluxim AG). The 650 nm long-pass filter used to block the LED light was purchased from ThorLabs. IMPLS measurements for the encapsulated film were collected by configuring the PAIOS system to measure the photodiode signal under IMPS mode.

Reference measurements were performed as described in the main text and shown in Figure S2. While the low amplitude signal indicates that the effect of light leakage is negligible at all frequencies, the system response becomes relevant at relatively high frequencies. This response arises from the combined effects of the photodiode and LED response times, the system controller, and the system readout.

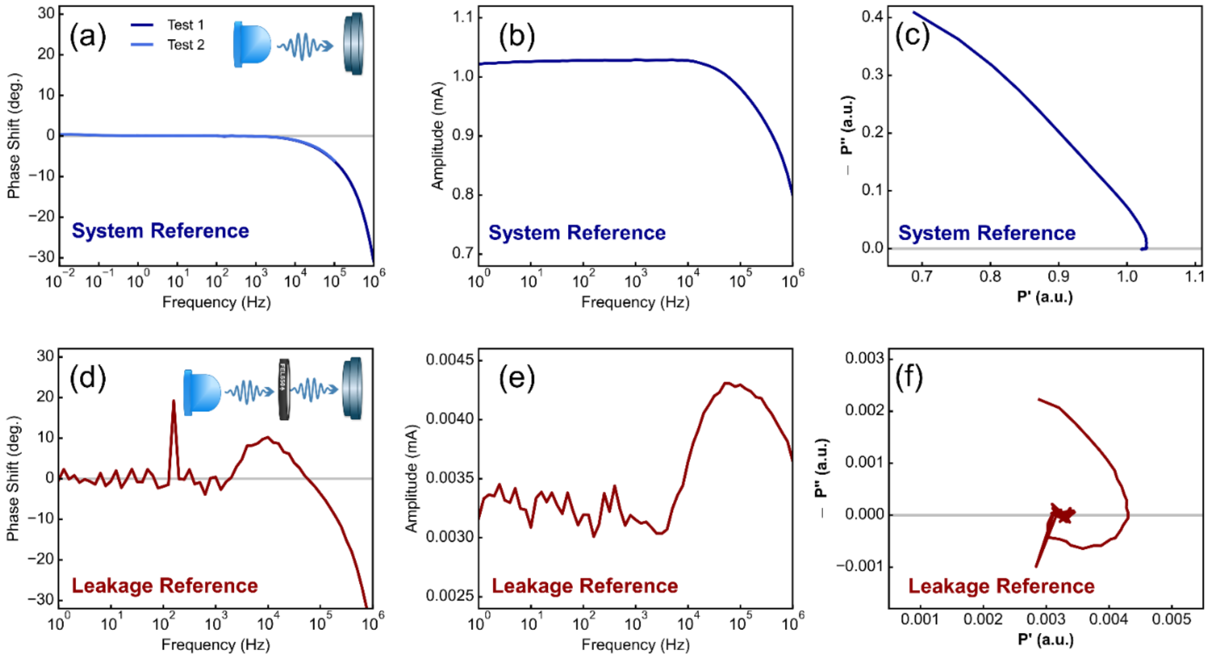

Figure S2: The top row represents the system reference measurement where the test configuration is depicted in the inset in (a). The results from the system reference are shown in blue where (a) shows the relative phase shift across two separate frequency ranges, (b) shows the corresponding amplitude and (c) is the generated Nyquist plot from these values. The bottom row represents the reference measurement to determine the influence of light leakage through the long-pass filter. The configuration of this reference is visualized with inset in (d). The relative phase shift, amplitude and corresponding Nyquist plot from the leakage reference measurement are shown in red in (d), (e) and (f), respectively.

The complete forward and reverse scan from the exemplary measurement shown in Figure 2 of the main text is presented in Figures S3a and S3b. Notably, the fast process was quenched when measuring the reverse scan (Figure S3a). This quenching is also visible in the amplitude plot, which does not recover during the frequency sweep reversal (Figure S3b). After keeping the sample in the dark overnight, IMPLS was re-conducted on the same spot under the same conditions. Interestingly, the PL amplitude not only recovered but exceeded its initial value, and the previously quenched fast process re-emerged (Figures S3c and S3d). These results suggest that the observed changes are due to a reversible state change in the material and not a consequence of permanent photodegradation.

We then investigated the influence of starting frequency and scan direction on the perovskite's IMPLS response. A new experiment was conducted, which included a pre-conditioning step in which the sample was illuminated with DC light for 2 minutes before the IMPLS scan was measured. The first measurement started at 1 MHz, sweeping down to 10 mHz and back up. After recovery in the dark, a second measurement began at 10 mHz and was swept upward. The comparison of these measurements is shown in Figure S4. The red curve (starting at 1 MHz) shows trends similar to those in Figure 2 of the main text, albeit with an amplitude enhancement at around 100 mHz. In contrast, the blue curve (starting at 10 mHz) shows no fast process in either direction. Furthermore, the PL amplitude of the slow process is reversible in the measurement starting at 10 mHz. The results indicate that one or more (possibly coupled) slow processes gradually quench the fast process under prolonged modulated light exposure.
In order to quantify the fast process, the IMPLS measurement must be conducted relatively quickly so that this fast process remains in a quasi-stable state. Figures S5, S7a and S7b show that the fast process remained quasi-stable in a shortened IMPLS scan, verifying that separate measurements and analyses of the fast and slow processes are required.

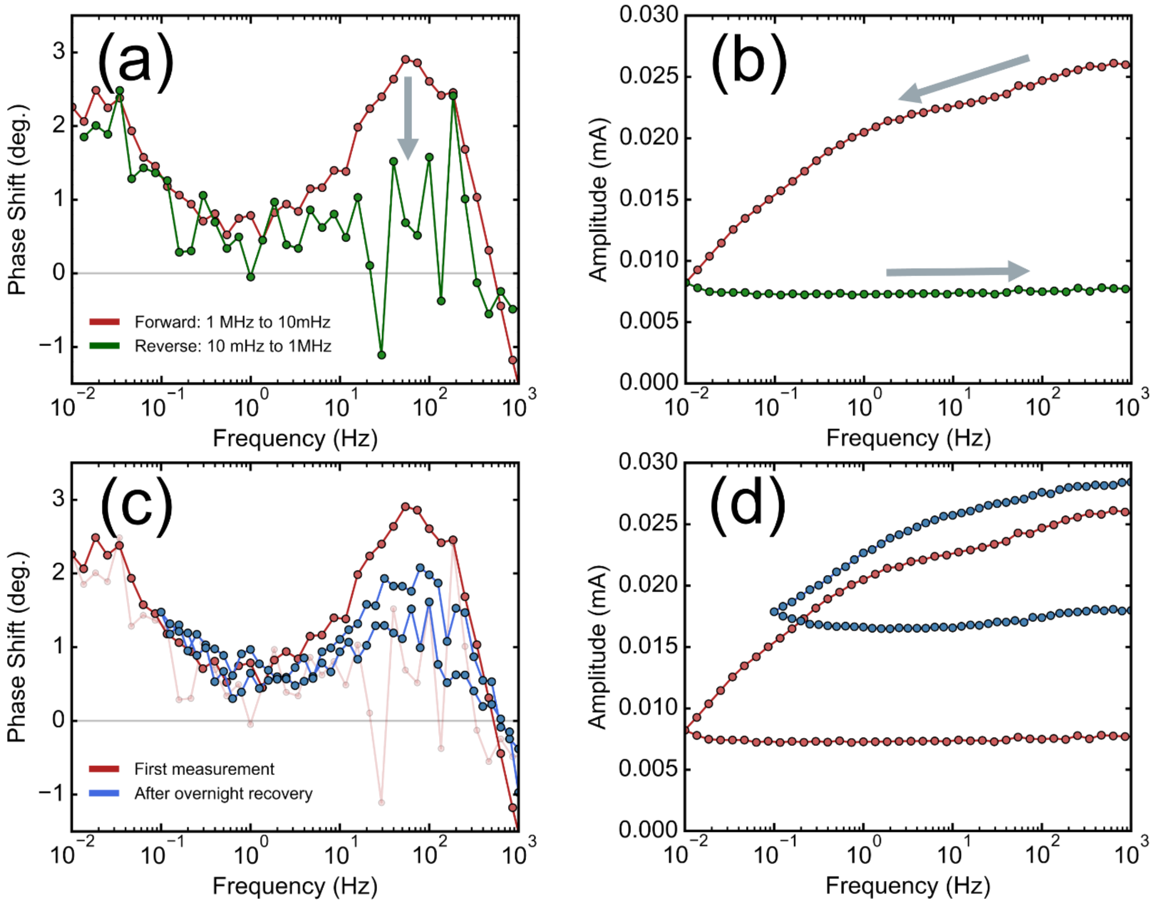

Figure S3: (a) The phase and (b) the amplitude for both the first half (red) and second half (green) of the IMPLS measurement presented in Figure 2 in the main text. As shown with the gray arrow in (a), the fast response is quenched between the forward and reverse sweep in this case. This is accompanied by the quenched PL amplitude as shown in (b). (c) The phase and (d) amplitude of the same sample spot, re-measured after keeping the sample in the dark overnight. The fast process is re-observed in the phase plot and is accompanied with an absolute PL amplitude enhancement.

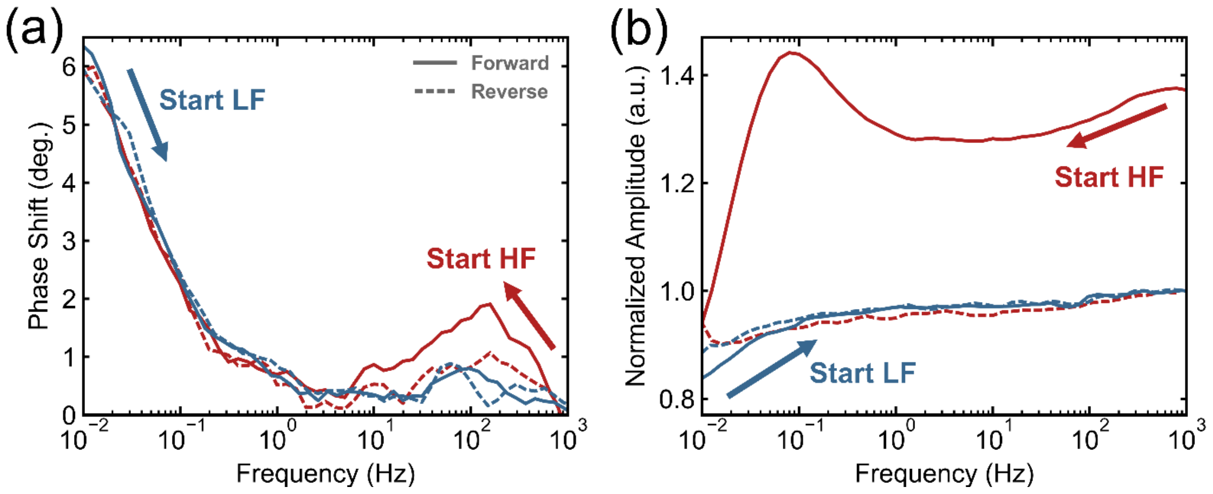

Figure S4: (a) Phase shift and (b) amplitude for two IMPLS measurements with different starting frequencies. The red curve in both panels represents the scan that begins at 1 MHz, with the solid red line showing the response during the first half of the scan (1 MHz to 10 mHz) and the dashed red line showing the response during the second half (10 mHz to 1 MHz). The blue curve represents the scan that begins at 10 mHz, with the solid blue line corresponding to the first half of the scan (10 mHz to 1 MHz) and the dashed blue line representing the second half (1 MHz to 10 mHz).

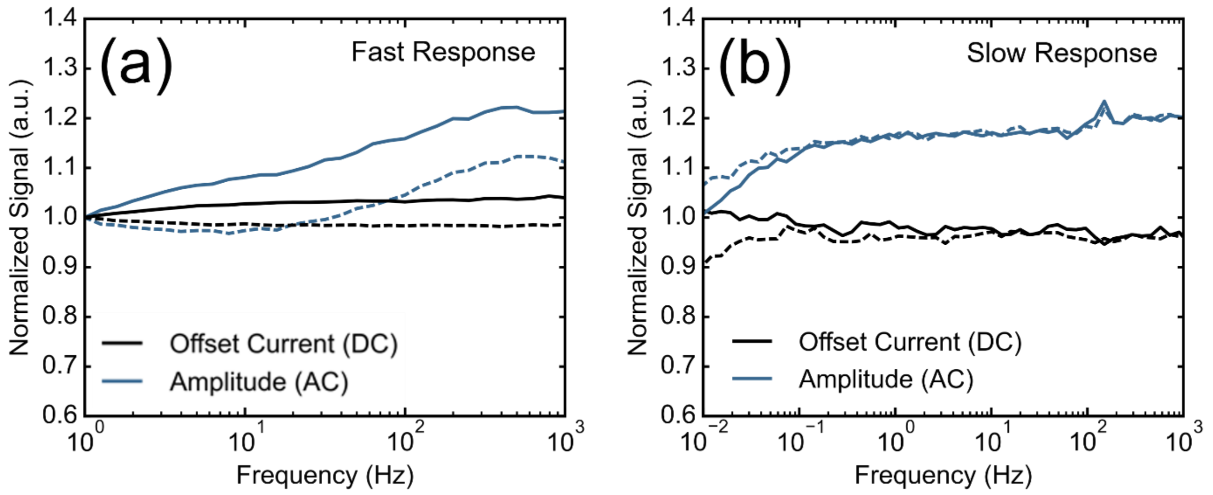

Figure S5: Comparison between the normalized AC (blue) and DC (black) current signals extracted for the (a) fast and (b) slow responses, corresponding to Figures 3 and 4 of the main text, respectively. The solid lines represent the first halves of the scans and the dashed lines represent the second halves. In both measurements, the DC signal does not deviate by more than 10% from its starting value, indicating that the processes remain in a quasi-stable state for the duration of the IMPLS measurements.

3. Optical Equivalent Circuit Modeling

Applying an OEC model is an effective, simple tool to fit the frequency-dependent rate equation to IMPLS data, where non-trivial, frequency-dependent processes can also be accounted for by including branches with their relevant circuit component proxies. The excess electronic minority carriers (Δn - which varies sinusoidally as a function of time during an IMPLS scan) corresponding to the OEC model described in this work is:

$$\frac{d\,\Delta n(t)}{dt} = G(t) - r_{rad}(t) - r_{Auger}(t) - r_{trap}(t) - r_{LF}(t) \qquad \textbf{S1}$$

Where $G(t)$ is the generation rate and r represents each of the different processes' recombination rates. $r_{LF}(t)$ is a term which accounts for all non-electronic processes that occur at lower frequencies (such as ionic migration and chemical reactions), which impact the carrier density. For sufficiently low frequencies, the fast electronic processes effectively behave as if in DC conditions. Then, this equation is simplified to:

$$\frac{d\,\Delta n(t)}{dt} = G - r_{HF} - r_{LF}(t) \qquad \textbf{S2}$$

Where only $r_{LF}(t)$ affects the frequency-dependent carrier density and thus the PL.

In principle, it is possible to separate these high frequency recombination processes with IMPLS provided that the system components have sufficiently high response times. In this case, the HF branch would then be separated into the three branches representing each of the electronic recombination processes (Figure S6a). Using Kirchhoff's law for parallel circuits, the corresponding resistors for each of these processes relate to R_{HF} by:

$$\frac{1}{R_{HF}} = \frac{1}{R_{rad}} + \frac{1}{R_{Auger}} + \frac{1}{R_{trap}} \qquad \textbf{S3}$$

Fitting at high frequencies thus allows for the extraction of the (modulated) PLQY, previously defined in Equation 3 of the main text.

This analysis ultimately means that when we are probing the slow process, the fast process is not relevant and can additionally be considered frequency-independent. Hence, the fast process is combined with the electronic processes in Figure 4a of the main text, and the analysis is simplified with the use of a single resistor. The converse is also true when we are measuring the fast process as in Figure 3a. However we emphasize that these separate analyses are not separate OECs. Rather, the schematics shown in Figures 3a and 4a of the main text are a simplification of the combined OEC which captures all of the electronic, fast and slow processes together. For completeness, this combined OEC is shown in Figure S6b.

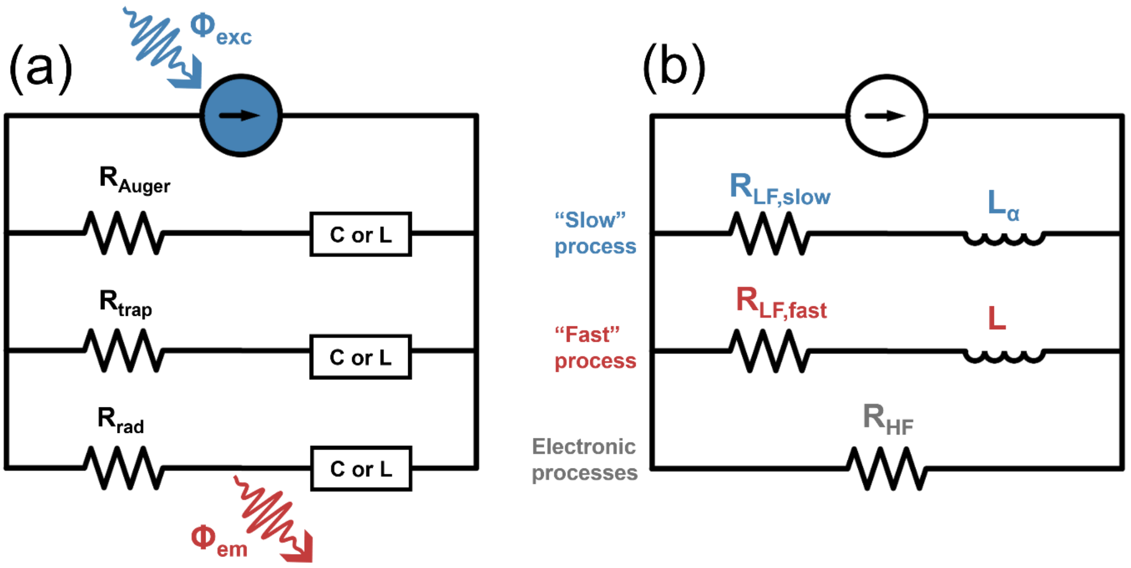

Figure S6: (a) A theoretical OEC model for when only electronic processes are considered. At sufficiently high frequencies, separate electronic responses (such as trap-assisted recombination) in principle can be resolved using a high-frequency adaptation of IMPLS combined with the appropriate OEC model to describe the data. As this OEC model is for exemplary purposes, only the influences of radiative, trap-assisted and Auger recombination are considered. The complex circuit components are exemplified with either an ideal capacitor or an ideal inductor (labelled as "C or L"), though other components may also be required to best fit and physically describe the electronic processes.
(b) The complete OEC model separating the fast and slow processes observed in this work.

Table S2: Extracted equivalent circuit parameters for the fast process using the simplified OEC shown in Figure 3a of the main text.

Direction	R_{HF} (a.u.)	R_{LF} (a.u.)	L (ms)	f_{char} (Hz)	τ_{char} (ms)
Forward	1	8.2±0.2	18±1	73±4	2.2±0.1
Reverse	1	6.6±0.1	13.2±0.6	79±4	2.0±0.1

Table S3: Extracted equivalent circuit parameters for the slow process using the OEC shown in Figure 4a in the main text.

Direction	R_{HF} (a.u.)	R_{LF} (a.u.)	L (s)	α (a.u.)	f_{char} (mHz)	τ_{char} (s)
Forward	1	1.9±0.1	121±8.4	0.65±0.01	2.5±0.3	63±6
Reverse	1	1.5±0.5	137±8.9	0.67±0.01	1.7±0.6	91±31

It is emphasized that the extracted values for R and L in Tables S2 and S3 do not correspond to physical electronic circuit components – rather, they simply correspond to fit parameters required to obtain the values of the characteristic frequency and lifetime in the OEC model fits.

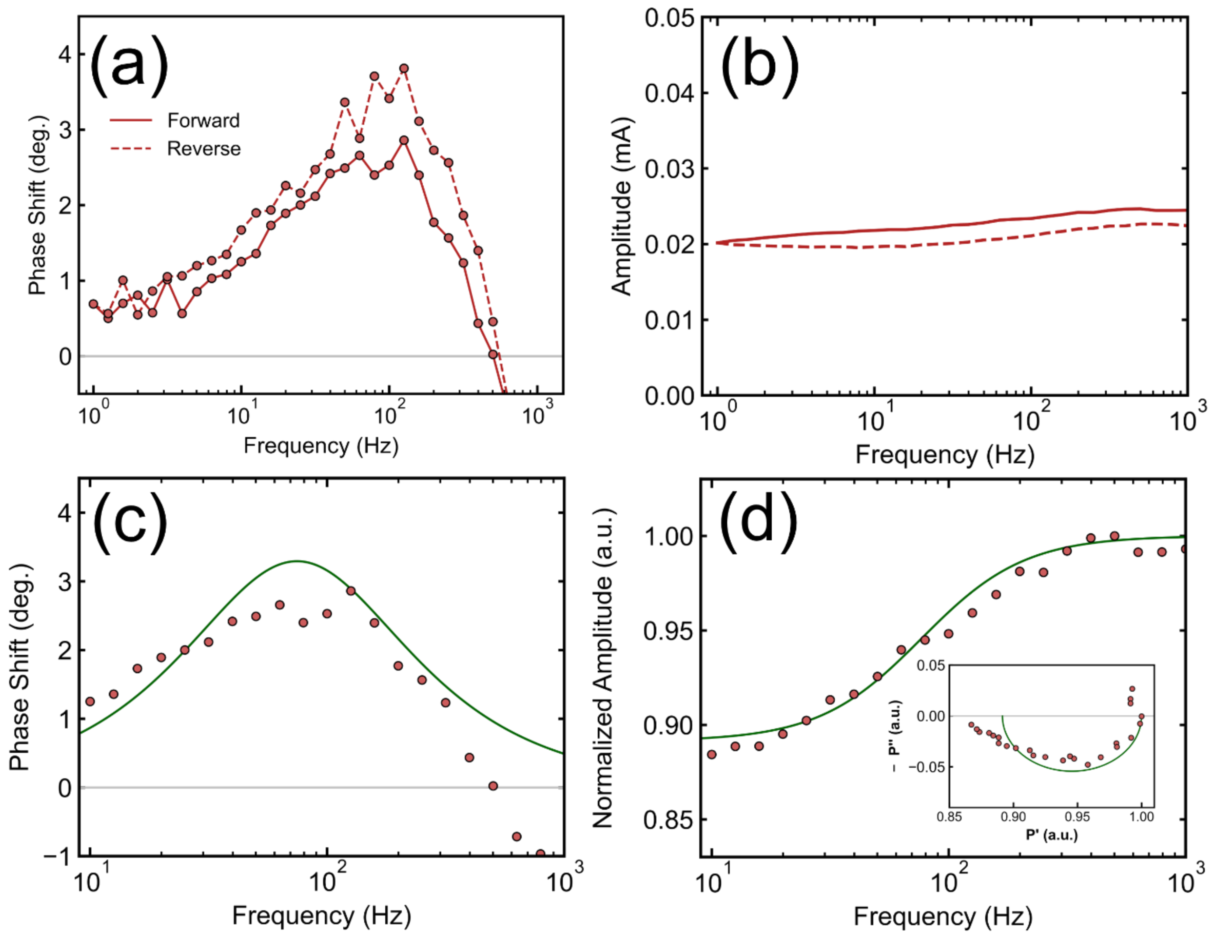

Figure S7: (a) The phase and (b) the amplitude for the IMPLS measurement sweeping only from 1 kHz to 1 Hz (solid line) and back (dashed line). (c) The data (red) and corresponding OEC model fit (green) for the phase of the forward scan. (d) The data and OEC model fit for the amplitude of the forward scan. Inset in (d): the OEC model fit to the corresponding Nyquist plot.

It is worth noting that the frequency at which the phase shift is at its maximum in the Bode plots does not necessarily correspond to the characteristic frequency of the process. This is because the characteristic frequency depends only on the process and is not coupled to any other effect in the system (as described in Equation 6 of the main text). However, the measured phase in general is related to all processes occurring in parallel:

$$\theta(\omega) = \arctan\frac{\mathrm{Im}|P_{\mathrm{Total}}|}{\mathrm{Re}|P_{\mathrm{Total}}|} \qquad \textbf{S4}$$

Where $\omega = 2\pi f$ and

$$\frac{1}{P_{\mathrm{Total}}} \propto \frac{1}{R_{\mathrm{HF}}} + \frac{1}{R_{\mathrm{LF}} + i\omega L} \qquad \textbf{S5}$$

As is often the case, the difference between using the OEC fitting and the peak in the Bode plot is small (79 Hz versus 86 Hz, respectively, in the case of Figure 3), but we use the more accurate OEC fitting value.

It is also worth further discussing the relevance of the fractional exponent term, alpha (α), that was required to fit the slow process in this work. Mathematically, α alters Equation S5 by:

$$\frac{1}{P_{Total}} \propto \frac{1}{R_{HF}} + \frac{1}{R_{LF} + i\omega^{\alpha} L^{\alpha}} \qquad \textbf{S6}$$

When α = 1, the process is described by a singular, well-defined relaxation time. There is likely no coupling or external factors occurring within in the system which alters such a process. This is similar to how trap-assisted recombination may be modelled using ECs in impedance spectroscopy. In the electrical analog, the process is effectively behaving as a perfect inductor. In IMPLS, we assume that ion Frenkel pair formation and annihilation would similarly follow this ideal behavior. When α = 0.5, the process is regarded to be entirely diffusive in nature and reflects mass transport limitations. This behavior aligns with that described by the Walburg element, which has previously been applied to model ion migration in perovskite devices in the past[4]. As shown in Table S3, α ranges from 0.64 to 0.68, which implies that the slow process we have measured is likely to be predominantly (but not perfectly) diffusive in nature. Simultaneous diffusion of different ionic species, grain boundaries, the thickness of the perovskite layer and reactions at the contact layers may impact the exact value for α. As we have speculated in the main text, the slow process may indeed be considered the combined effect of vacancy diffusion followed by further Frenkel pair formation, which in turn would ultimately place α somewhere between 0.5 and 1.

All OEC fitting was conducted using the *impedance.py* package in Python[5]. The quality of each of these fits were assessed using the coefficient of determination, R^2, which is tabulated for the real and imaginary components of the OEC fits in Table S4. R^2 of the imaginary component for the fast process is notably smaller than the slow component's imaginary fit, which we attribute to the onset of the system response in the fast process (occurring near 1 kHz). This system response is not present in the slow process frequency range which allows for a much higher R^2 for the imaginary component fits. However, as it is clear where the phase shift peaks in the Bode plots in Figure 3c and in Figure S7c, we can confirm that the fits still are well assessed for the given data.

Table S4: The coefficient of determination (R^2) for each of the four OEC fits.

	**Fast (Foward)**	**Fast (Reverse)**	**Slow (Forward)**	**Slow (Reverse)**
Real R^2	0.9733	0.9877	0.9963	0.9492
Imaginary R^2	0.5687	0.6479	0.9891	0.9758
Combined R^2	0.9390	0.9440	0.9937	0.9651

Sensitivity analyses in general show that the model is more sensitive to changes in R_{LF} than in L; this is exemplified in Figure S8 which shows the sensitivity analysis results for the forward scan fit of the fast process. The combined R^2 deviates by 3.3% when the value for L is reduced by 20%, and by only 2.2% when L is increased by 20%. However the combined R^2 deviates by 13.3% when R_{LF} is reduced by 20%, and 7.1% when R_{LF} is increased by 20%.

The maxima shown in both Figures S8a and S8b further verify that the obtained fits are optimized for the IMPLS data.

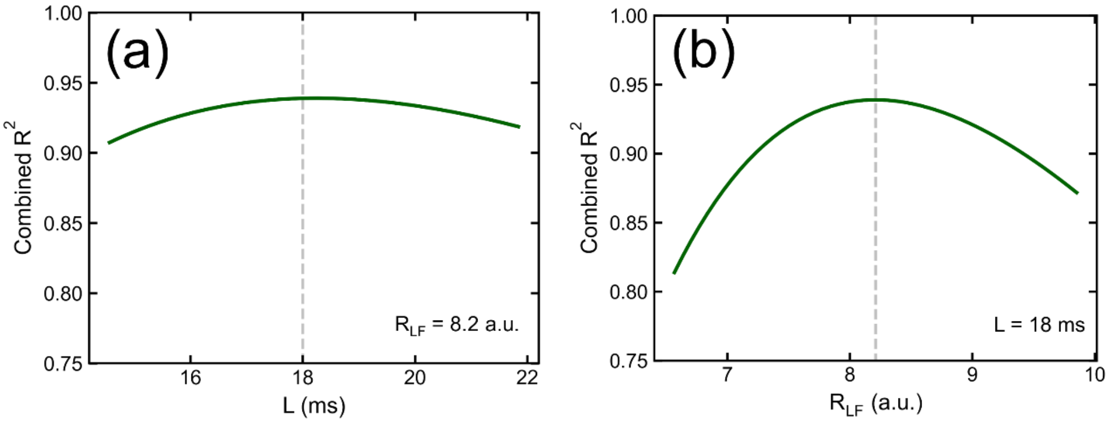

Figure S8: Sensitivity analysis for the forward scan of the fast process. (a) The sensitivity of the combined residuals when R_{LF} is fixed at 8.2 a.u., and L is swept ± 20% of its fitted value of 18 ms (shown with the gray dashed line). (b) The sensitivity of the combined residuals when L is fixed at 18 ms, and R_{LF} is similarly swept ± 20% of its fitted value of 8.2 a.u. (similarly shown with the gray dashed line).

4. Fabrication and Characterization of Perovskite Devices

Pre-patterned (2.5×2.5 cm^2, 15 Ω/sq) ITO substrates (Psiotec, UK) were sonicated for 10 minutes subsequently in acetone, 3% Hellmanex solution in deionized (DI) water, DI-water and isopropanol in order to clean them. After an oxygen plasma treatment (4 min, 120 W), the substrates were transferred to a N_2-filled glovebox.

To deposit the hole transport layer, 60 µL of PTAA (Poly-[bis-(4-phenyl)- (2,4,6-trimethylphenyl)-amin]) solution (Sigma-Aldrich, 1.75 mg/mL in toluene) was spin-coated onto the substrates at 6000 rpm for 30 seconds with a ramp of 2000 rpm/s. After 10 min annealing on a hotplate at 100 °C, the films were cooled down to room temperature. 60 µL of PFN-Br (Poly(9,9-bis(3'-(N,N-dimethyl)-N-ethylammoinium-propyl-2,7-fluorene)-alt-2,7-(9,9-dioctylfluorene))dibromide) solution (1-Material, 0.5 mg/ml in methanol) was deposited on top of the PTAA layer dynamically at 4000 rpm for 30 s.

The solutions for the perovskite active layers were prepared in a similar manner to the bare films: 1.2 M $FAPbI_3$ solution was prepared by dissolving FAI and PbI_2 in DMF:DMSO (4:1 volume ratio) which contains a 10%-molar excess of PbI_2. The 1.2 M $MAPbBr_3$ solution was made by dissolving MABr and $PbBr_2$ in DMF:DMSO (4:1 volume ratio) which contains a 10 %-molar excess of $PbBr_2$. The solutions were stirred overnight at room temperature. By mixing these $FAPbI_3$ and $MAPbBr_3$ solutions in a ratio of 83:17 and adding 42 µL 1.5M CsI

solution in DMSO to 958 µL of the 83:17 mixture, the desired solution was obtained.
The triple cation perovskite films were prepared by depositing 120 µL and spin-coating at 4000 rpm for 400 s at a ramp of 1334 rpm/s. 10 s after the start of the spinning process, the spinning substrate was washed with 300 µL ethylacetate for approximately 1 s (the anti-solvent was deposited in the centre of the film). The perovskite film was then annealed at 100 °C for 1 h on a preheated hotplate.
After annealing, the samples were transferred to an evaporation chamber where fullerene C60 (25 nm), 2,9-Dimethyl-4,7-diphenyl-1,10-phenanthroline BCP (8 nm) and copper (100 nm) were deposited under vacuum (p = 10^{-7} mbar). The overlap of the copper and the ITO electrodes defined the active area of the pixel (6 mm^2).

The *JV* curves of these devices were measured under an N_2 atmosphere using a Keithley 2401 SMU and an G2V Pico LED solar simulator. The scan rate for the *JV* curve shown in Figure 6a of the main text was 0.1 V/s. The modulated *IV* curves (Figure 5b of the main text), the IMPS and IMVS scans (Figures 5c,d) and the scan-rate dependent *IV* curves were measured using an Agilent B2902A SMU with 450 nm blue LED (Cree LED). The devices were left unmasked for these measurements and the illumination power density was approximately 120 mW/cm^2.
IMPLS measurements of the solar cell (Figure S10) were obtained using the WiTEC alpha300 SR confocal imaging microscope acting as the PL detector and with the Agilent B2902A SMU coupled to a 450 nm blue LED (Cree LED) to supply the DC and AC light signal.

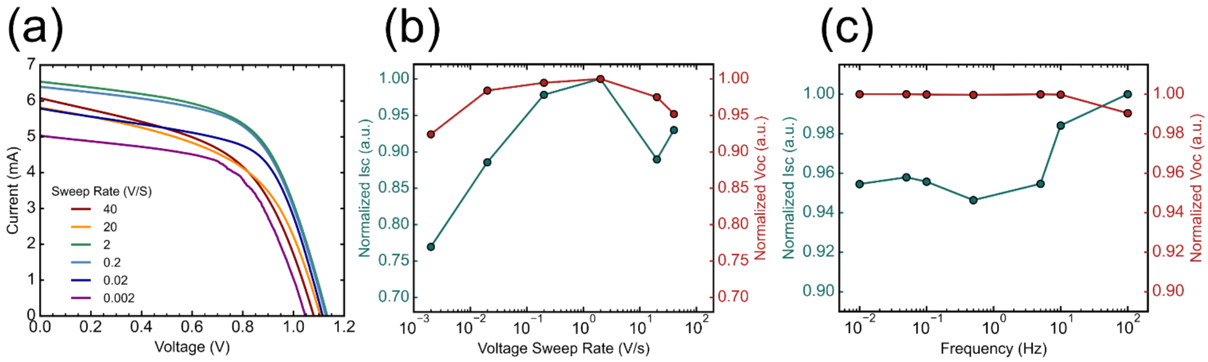

Figure S9: (a) Measured *IV* curves at different voltage scan rates, where the rates are shown in the legend. (b) The normalized I_{SC} (left) and normalized V_{OC} (right) as a function of the voltage scan rate, extracted from the device *IV* curves shown in panel (a). (c) The normalized I_{SC} (left) and normalized V_{OC} (right) as a function of AC modulation frequency for the data shown in Figure 5b of the main text. Panels (b) and (c) highlight that the current is more strongly influenced than the voltage in both the voltage scan rate measurements and light modulation frequency measurements.

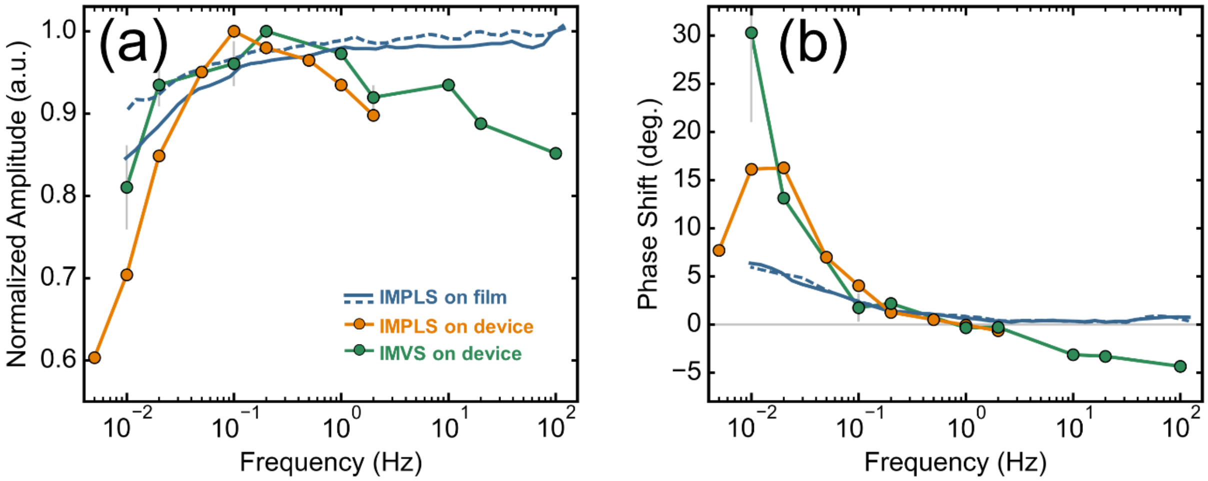

Figure S10: (a) Measured IMPLS amplitude for the solar cell (orange) overlaid with the IMPLS amplitude of the encapsulated film (blue) and the IMVS amplitude for the same device (green). (b) The corresponding phase shifts for the same IMPLS and IMVS measurements.

References

[1] Gillespie, S. C.; Gautier, J.; van der Burgt, J. S.; Anker, J.; Geerligs, B. L.; Coletti, G.;Garnett, E. C. Silicon-Inspired Analysis of Interfacial Recombination in Perovskite Photovoltaics. *Advanced Energy Materials* **2024**, *14*, 2400965.

[2] Alvarez, A. O.; Ravishankar, S.; Fabregat-Santiago, F. Combining modulated techniques for the analysis of photosensitive devices. *Small Methods* **2021**, *5*, 2100661.

[3] Sánchez, R. S.; Villanueva-Antolí, A.; Bou, A.; Ruiz-Murillo, M.; Mora-Sero, I.; Bisquert, J. Radiative Recombination Processes in Halide Perovskites Observed by Light Emission Voltage Modulated Spectroscopy. *Advanced Materials* **2023**, *35*, 2207993.

[4] Peng, W.; Aranda, C.; Bakr, O.M.; Garcia-Belmonte, G.; Bisquert, J.; Guerrero, A. Quantification of Ionic Diffusion in Lead Halide Perovskite Single Crystals. *ACS Energy Letters* **2018**, *3*, 1477 – 1481.

[5] Murbach, M.D.; Gerwe, B.; Dawson-Elli, N.; Tsui, L.J. impedance.py: A Python package for electrochemical impedance analysis. *Journal of Open Source Software* **2020**, *5(52)*, 2349.
